# Improvement in diagnosis of breast tumour using ultrasound elastography and echography: A phantom based analysis

**DOI:** 10.2349/biij.5.4.e30

**Published:** 2009-10-01

**Authors:** K Kumar, ME Andrews, V Jayashankar, AK Mishra, S Suresh

**Affiliations:** 1 Department of Electrical Engineering, IIT Madras, Chennai, India; 2 Department of Chemistry, IIT Madras, Chennai, India; 3 Mediscan Systems, Chennai, India

**Keywords:** Elastography, classifier, ROC, texture, tissue phantom

## Abstract

Due to the isoechoic nature of lesions and their poor contrast with neighbouring tissue, a lesion may remain undetected in ultrasound B mode imaging for cancerous tissue. Imaging of the elastic properties of tissue provides new information which is collateral to tissue pathology. This study provides quantitative analysis of improvements in tumour diagnosis when the ultrasound B mode imaging is combined with elastography. Quantification was based on the textural parameters measured from the ultrasound B mode image and strain measured from the elastogram. The ability of a parameter to discriminate between diseased cases and normal cases was evaluated using receiver operating characteristic (ROC) analysis. Polyacrylamide gel based tissue mimicking phantoms with embedded inclusions of varying stiffness were used for the analysis.

## INTRODUCTION

Particularly when the task at hand is to differentiate between malignant tumours and other possibly benign conditions, non-invasive identification of a breast tumour is a challenging area of medical diagnosis. Prior to the advent of diagnostic imaging, the oldest method of detection involved palpation. While palpation is understandably simple, it is nevertheless just a qualitative assessment and can only be applied to superficial organs. Malignant tumours feel harder than benign ones and this physical property is related to their coefficient of elasticity. The elasticity of soft tissues depends on their molecular building blocks (fat, collagen, etc.) and on their microscopic and macroscopic structural organisation of these blocks [1-3]. Pathological changes are generally correlated with changes in tissue stiffness as well. In many cases, the lesion may not possess sufficient echo graphic properties and therefore it avoids detection using normal B mode ultrasound. As the echogenecity and the stiffness of tissue are generally uncorrelated, it is expected that imaging tissue stiffness will provide new information that is related to tissue structure and or pathology [3].

Elastography is a method of estimating the elastic properties of biological tissues [3-7]. Elastograms are obtained by comparing ultrasound echo data obtained before and after a slight compression of the tissue. The results of the comparison are displayed as an image, where soft areas appear white and hard areas appear black. Garra et al. [8] used ultrasound elastography for the examination of breast lesions using a 5 MHz linear array transducer. The elastogram and corresponding sonogram were evaluated by a single observer for lesion visualisation, relative brightness and margin definition. The mean depth and width of the lesion on the elastogram and sonogram were measured and used as parameter for differentiating benign and malignant lesions. Andrej et al. [9] and Rago et al. [10] performed real time elastography on the thyroid lesions. All elastograms were evaluated for the lesion visibility, relative brightness, and margin regularity and definition by using a four point scale. Zhi et al. [11] evaluated the capability of ultrasound elastography in differentiating between benign and malignant lesions of the breast, and compared this with conventional sonography and mammography. They concluded that ultrasound elastography is superior to conventional sonography, and furthermore is superior or equal to mammography in differentiating between benign and malignant lesions in the breast. By combining ultrasound elastography and sonography, accuracy of detection can be improved greatly, while retaining the potential to reduce unnecessary biopsy.

None of the above studies attempted a quantitative analysis of the improvement in classification accuracy when ultrasound elastography is combined with echography. The main focus of this study is to quantify the improvement in diagnosis of tumours by combining the ultrasound B mode imaging with elastography. Quantification is based on the textural parameters measured from the ultrasound B mode image and strain measured from the elastogram.

## MATERIALS AND METHODS>

### Phantom preparation

Polyacrylamide gel based tissue mimicking phantoms [12] were designed for study and analysis. The polymerisation of acrylamide was carried out in deionised water at room temperature using bisacrylamide as the cross linker, and mmonium persulfate (APS) and tetramethylethylenediamine (TEMED) as a pair of redox initiators. Fine particles of titanium dioxide were used to control the echogenecity of the gel. The elasticity of the phantom was varied by adjusting the acrylamide concentration so that it covered a wide range of normal to pathological tissue stiffness.

Acoustic properties of the phantoms were measured using the pulse-echo-transmit-receive principle, with a single element transducer of 5 MHz centre frequency. Young's modulus of the sample was measured using the unconfined compression test. A variable compression load was applied on the sample and corresponding deformation is measured. From these data, stress-strain characteristics of the sample were plotted and the modulus was measured.

Two categories of phantoms were designed with embedded inclusions of varying stiffness. In the first category the elasticity was maintained constant and echogenecity was varied, thus producing isoechoic (indistinguishable from surrounding tissue), hyperechoic (appearing brighter than surrounding tissue), hypoechoic (appearing darker than surrounding tissue) and anechoic lesions (those without echoes). In the second category echogenecity was kept constant and elasticity was varied.

**Figure 1:**
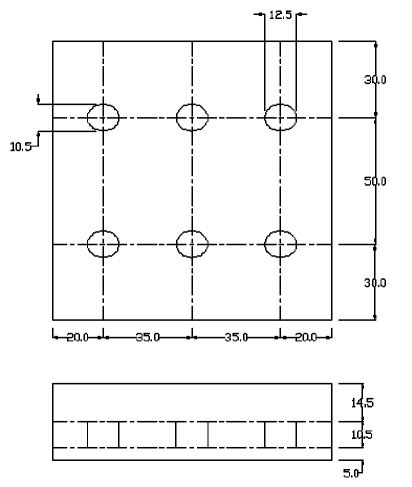
Plan and elevation of a typical phantom with six embedded inclusions of varying stiffness. (Phantom details: prepared with 8% acrylamide, 0.06% initiator and 40% TiO2 Concentration of acrylamide in inclusions was varied from 6% to 20%. Concentration of TiO2 and initiator was same in all the inclusions and is equal to 40% and 0.06% respectively). All measurements are in mm.

**Figure 2:**
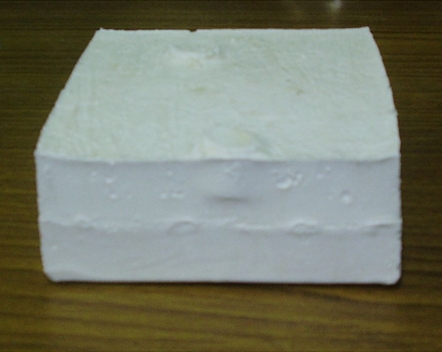
External view of the phantom.

The plan and elevation of a typical phantom from the second category is shown in Figure 1. The external view of the phantom is shown in Figure 2. The ultrasound B mode and elastographic behaviour of these lesions was analysed using image processing methods.

### Image acquisition

Ultrasound B mode and elastograms were acquired using Siemens ACUSON Antares [13], at Mediscan System, Chennai. Linear array transducer VF 7-3 with a probe frequency of 5 MHz was used. Initially, B mode image of the region of interest (ROI) was obtained and then a slight compression was applied by pressing the transducer on the surface. Built- in software present in the system generated the elastogram by comparing the pre-compressed and post-compressed RF signals, and displayed these adjacent to the B mode image for comparison and more accurate diagnosis.

### Tissue characterising parameters

Texture parameters were measured to quantify the B mode images [14-15]. The first order parameters used in this study were mean value, variance, skewness, kurtosis, energy and entropy, and accounted for variations in intensity without spatial reference. These parameters were measured by computing the image histogram, the shape of which provided many clues as to the character of the image. Second order texture parameters were extracted from the normalised co-occurrence matrices [16] and include angular second momentum, correlation, inertia, absolute value, inverse difference, entropy and maximum probability. While first order texture parameters cannot completely characterise texture, second order texture parameters account for changes in the spatial arrangements of intensities.

### Quantitative analysis of parameters

The ability of a test to discriminate between diseased cases and normal cases was based on receiver operating characteristic (ROC) analysis. The ROC curve represents a plot describing the classifier's true positive (TP) detection rate against its false positive (FP) rate [17-18]. In medical imaging the TP rate is commonly referred to as sensitivity and (1.0 – FP rate) is referred to as specificity.

The histograms of each parameter were calculated to obtain the distribution of parameter values for both normal and tumour groups. Specificity (S_p_) and sensitivity (S_e_), were determined as:

(1) $$S_{e}\;=\;\frac{TP}{FN\;+\;TP}\;S_{p}\;=\;\frac{TN}{TN\;+\;FP}$$

where *TN* is true negative and *FN* is false negative.

By changing the threshold, TP and FP rate can be varied. Plotting sensitivity against specificity, for different threshold values, ROC curve can be obtained. The area under the ROC curve (A_ROC_) is an accepted method of comparing classifier performance, and a perfect classifier ought to have a TP rate of 1.0 and a FP rate of 0.0, resulting in A_ROC_ of 1.0. Values of 0.5 < A_ROC_ ≥ 1 indicate the potential of the respective parameters to differentiate between the existence and absence of tumours.

### Bayesian classifier

The probability that a sample belongs to class C_i_, given that it has a feature value x denoted by P(C_i_/x) can be computed as [19]:

(2) $$g_{i}\left(x\right)\;=\;P\left(C_{i}/x\right)\;=\;\frac{p\left(x/C_{i}\right)P\left(C_{i}\right)}{\sum_{j=1}^{C}p\left(x/C_{j}\right)P\left(C_{j}\right)}$$

where p(x/C_i_) is the conditional probability of obtaining feature value x given that sample is from class C_i_, P(C_i_) is the prior probability that a random sample is a member of class C_i_ and C is the total number of classes. For a two dimensional case, the normal density function can be written as:

(3) $$p\left(x\right)\;=\;\frac{1}{2\pi \left|C_{v}\right|}e^{\left[-\frac{1}{2}{\left(x-\mu \right)}^{t}C_{v}{}^{-1}\left(x-\mu \right)\right]}$$

where C_v_ is the covariance matrix, μ is the mean vector, |C_v_| and C_v_^-1^ are the determinant and inverse of covariance matrix respectively.

A Bayesian classifier can be trained by determining the mean vector, and the covariance matrices for the normal and tumour classes from the training data. For the training corresponding to Class 1 (tumour) and Class 2 (normal) data, mean vector and covariance matrix were calculated separately. The parameters with highest values of A_ROC_ were chosen as the best performing parameters. Using these parameters, a discriminant function for a two class case can be defined as:

(4) $$g\left(x\right)\;=\;g_{1}\left(x\right)\;-\;g_{2}\left(x\right)$$

where g_1_(x) and g_2_(x) are discriminant functions corresponding to two classes. We use the following decision rule: decide class 1 if g(x) > 0; otherwise decide class 2. [19]

## RESULTS

**Figure 3:**
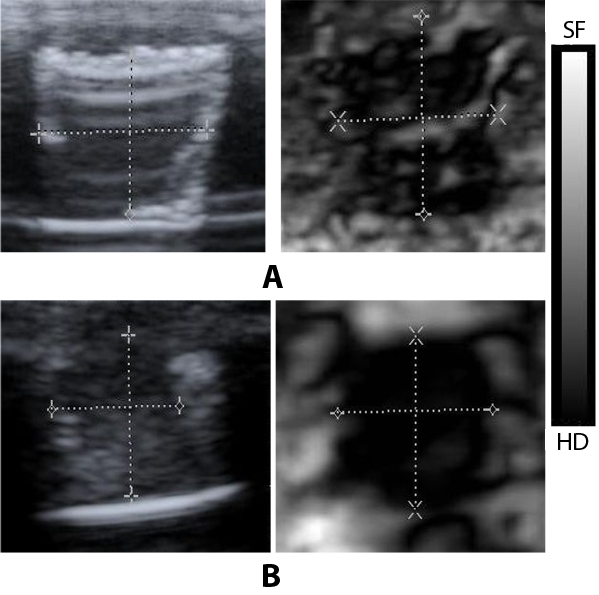
Ultrasound B mode image and elastogram of two solid lesions. Both the lesions appear hyperechoic in B mode but their characteristics are different in elastogram. (a) is less stiffer than (b). In elastogram black region represents hard (HD) and white region represents soft (SF). Line markings shows the width and depth of the lesion.

**Figure 4:**
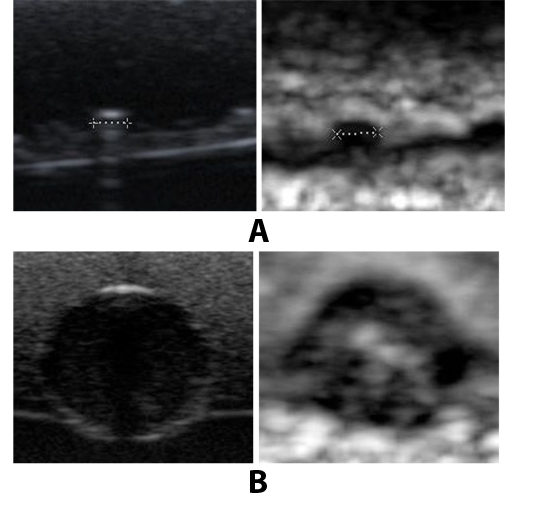
Ultrasound B mode image and elastogram of two lesions. (a) Very small hyperechoic lesion in B mode and hard lesion in elastogram. (b) Anechoic lesion in B mode and a Bull’s eye appearance in elastogram, a typical elastographic appearance of cystic lesion.

**Figure 5:**
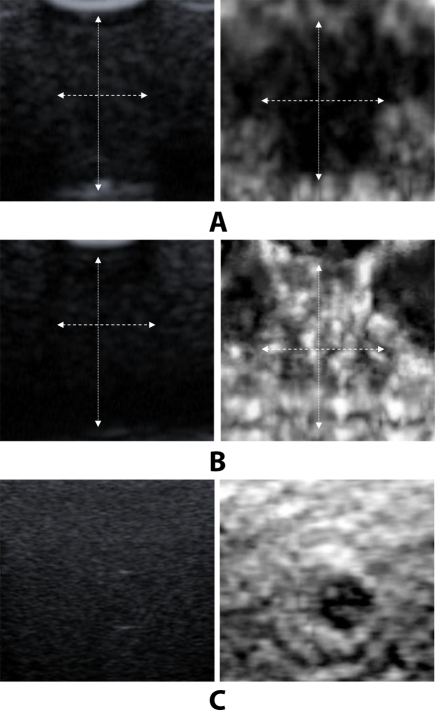
Ultrasound B mode image and elastogram of three solid lesions. Lesions appear isoechoic in B mode but their characteristics are different in elastogram. (a) and (c) are stiffer and (b) is soft. Arrow markings show the width and depth of the lesion.

The acoustic properties of the phantoms used for the analysis are shown in the Table 1 along with corresponding parameters for human tissue [20-21]. Figures 3, 4 and 5 show the ultrasound B mode and elastogram of the different lesions analysed.

**Figure 6:**
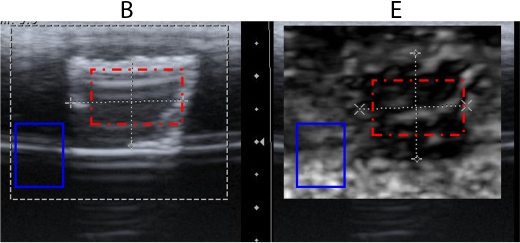
Ultrasound B mode image (B) and elastogram (E) of a hyperechoic lesion. Rectangular region marked in red (dashed line) in B represents the lesion in B mode image selected for parameter measurement and region marked in blue (solid line) is the region in surroundings selected for parameter measurement. Rectangular region marked in red (dashed line) in E represents the lesion in elastogram selected for average strain measurement and region marked in blue (solid line) is the region in surroundings selected for average strain measurement.

They are hyperechoic (Figure 3), anechoic (Figure 4b) and isoechoic (Figure 5) lesions respectively. In the elastogram , soft areas are represented by white and hard areas are represented by black. Figure 6 shows the areas on the B mode image and the elastogram for the measurement of parameters.

**Table 1:** Acoustic parameters of the designed phantom with corresponding parameters for human tissue [20], [21] for comparison

**Parameters**	**Human Tissue**	**Values Obtained**
Velocity (m/s)	1490 – 1610 (mean value: 1540)	1529.90
Attenuation (db/MHz-cm)	0.4 – 2.0 (mean value: 0.7)	0.66
Acoustic Impedance × 10^-6^ (kg/m^2^-s)	1.58 – 1.70 (mean value: 1.63)	1.60

From the designed phantoms, a total of 28 images from the embedded inclusions were obtained. Separation of normal regions and tumour regions was based on the Young's modulus of the inclusion, which in turn was controlled by the concentration of monomers. An inclusion with a Young's modulus of greater than 60 kPa was considered as a stiffer region and therefore deemed to be a tumour. Based on this criterion, among 28 inclusions, 7 were selected as normal, and the remaining 21 as tumour regions.

ROC analysis was performed for all 14 parameters, and finally parameters with A_ROC_ > 0.5 were used as input to the classifier. From the group of first order texture parameters, mean (M), skewness (SK) and entro (ENT) were used. The parameters used from the second order texture parameters were angular second momentum (ASM) and maximum probability (max_prob). In addition, average strain (STR) estimated from the elastogram was also included.

Results of the parameter evaluation using A_ROC_ are shown in Table 2. The total number of cases used for training was 5 normal and 8 tumours. Classification results with and without the average strain parameter (STR) are shown in the Table 3.

**Table 2:** Performance of selected parameters

**Parameter**	**M**	**SK**	**ENT**	**ASM**	**max_prob**	**STR**
AROC	0.59	0.51	0.56	0.58	0.52	0.73

**Table 3:** Classification accuracy with and without strain parameter

**Parameters considered**	**Classification accuracy**
Without STR	66.7% (10/15)
With STR	86.6% (13/15)

## DISCUSSION

The acoustic parameters of the phantoms designed in this study closely matched those of human tissue (Table 1). The lesions were clearly distinguishable in the elastogram, even though they appear almost similar in B mode. These results are clearly evident from the images shown in Figures 3, 4 and 5. In this study, along with average strain estimated from the elastogram, several tissue characterising parameters were used, all estimated from ultrasound B mode images. The ability of each parameter in differentiating between normal regions and tumour regions has been analysed. Class 1 included lesions whose Young's modulus is greater than 60 kPa; Class 2 included softer regions.

ROC analysis confirmed that the use of a single parameter is not recommended. And it has been shown that using high performance parameters collectively in the classification will produce an accuracy of 86.7%.

## CONCLUSION

Echogenicity and elasticity of the phantoms are suitably controlled to obtain normal and pathological lesions in the phantoms. An increase in the classification accuracy was achieved following the inclusion of the average strain parameter as an additional input to the classifier. Elastography may thus be considered as one of the available diagnosis tool for improving the accuracy of the diagnosis of breast and thyroid tumours.
